# Reimagining procedural distress as a candidate quality domain in pediatric emergency medicine

**DOI:** 10.3389/fped.2026.1841168

**Published:** 2026-06-24

**Authors:** Xiao-Tian Xu, Han Chen, Qian-Nan Ruan, Wen-Jing Yan

**Affiliations:** 1 Emergency Department, Third Affiliated Hospital of Wenzhou Medical University, Wenzhou, China; 2 School of Mental Health, Wenzhou Medical University, Wenzhou, China; 3 Zhejiang Provincial Clinical Research Center for Mental Health, The Affiliated Kangning Hospital of Wenzhou Medical University, Wenzhou, China

**Keywords:** comfort positioning, neurodivergence, pediatric emergency medicine, physical restraint, procedural distress, quality improvement, trauma-informed care

## Abstract

Over the past three decades, pediatric emergency medicine (PEM) has successfully championed the management of nociceptive pain, cementing it as the “fifth vital sign.” However, a clinical paradox persists: medical procedures that are adequately anesthetized against somatic pain may still be psychologically traumatic when procedural distress is unmanaged. In this Perspective, we argue that procedural distress should be reframed from an accepted operational byproduct to a modifiable and potentially preventable harm. We propose that it should be considered a candidate quality domain in pediatric emergency medicine. The framework distinguishes anticipatory fear screening from retrospective auditing of severe distress events and outlines operational thresholds for documentation and quality improvement. We also present an efficiency-chain model as a hypothesis-generating framework, not as an established causal pathway, and call for prospective validation that accounts for clinical confounders. By establishing a tiered classification for physical stabilization, acknowledging safety caveats, and institutionalizing the “clinical pause,” healthcare systems can better protect the psychological integrity of vulnerable children, including neurodivergent patients.

## 1 Introduction

Over the past three decades, pediatric emergency medicine (PEM) has achieved a monumental milestone by firmly establishing pain as the “fifth vital sign" (1). This necessary paradigm shift revolutionized clinical practice globally, compelling healthcare systems to transition away from historical models that vastly underestimated pediatric nociception. Today, the routine integration of standardized, age-appropriate scales for assessing physiological pain, coupled with the aggressive implementation of targeted pharmacological interventions (such as topical anesthetics, intranasal analgesics, and structured procedural sedation protocols) is widely recognized as the fundamental standard of care in pediatric emergency departments (EDs) (2).

However, lurking in the shadow of this biomedical success is a complex clinical paradox: a medical procedure that is adequately anesthetized from a strictly neurobiological standpoint frequently remains deeply traumatic psychologically. The prevailing clinical focus on neutralizing somatic pain intensity has inadvertently masked a critical operational and ethical blind spot in acute pediatric care—the pervasive phenomenon of procedural distress (3). By appropriately treating the biological signals of pain while largely under-addressing the psychological manifestations of fear and loss of control, modern pediatrics risks mitigating physical suffering at the expense of iatrogenic psychological trauma.

In this Perspective, we argue for transitioning beyond unidimensional pain scores to recognize procedural distress not as an unavoidable behavioral nuisance, but as a highly modifiable clinical state. Rather than proposing rigid Key Performance Indicators (KPIs) that risk unintended clinical consequences or institutional gaming, we propose a conceptual framework for operationalizing procedural distress and physical restraint as candidate quality domains. We outline actionable definitions for auditing, while explicitly acknowledging the complex realities, resource constraints, and safety imperatives inherent to the acute care environment.

Yet, the approach of unfamiliar clinicians and instruments can trigger marked fear, autonomic arousal, and loss of behavioral control. Under operational pressure in a high-acuity environment, the clinical team may resort to physical restraint.

**Figure 1:**
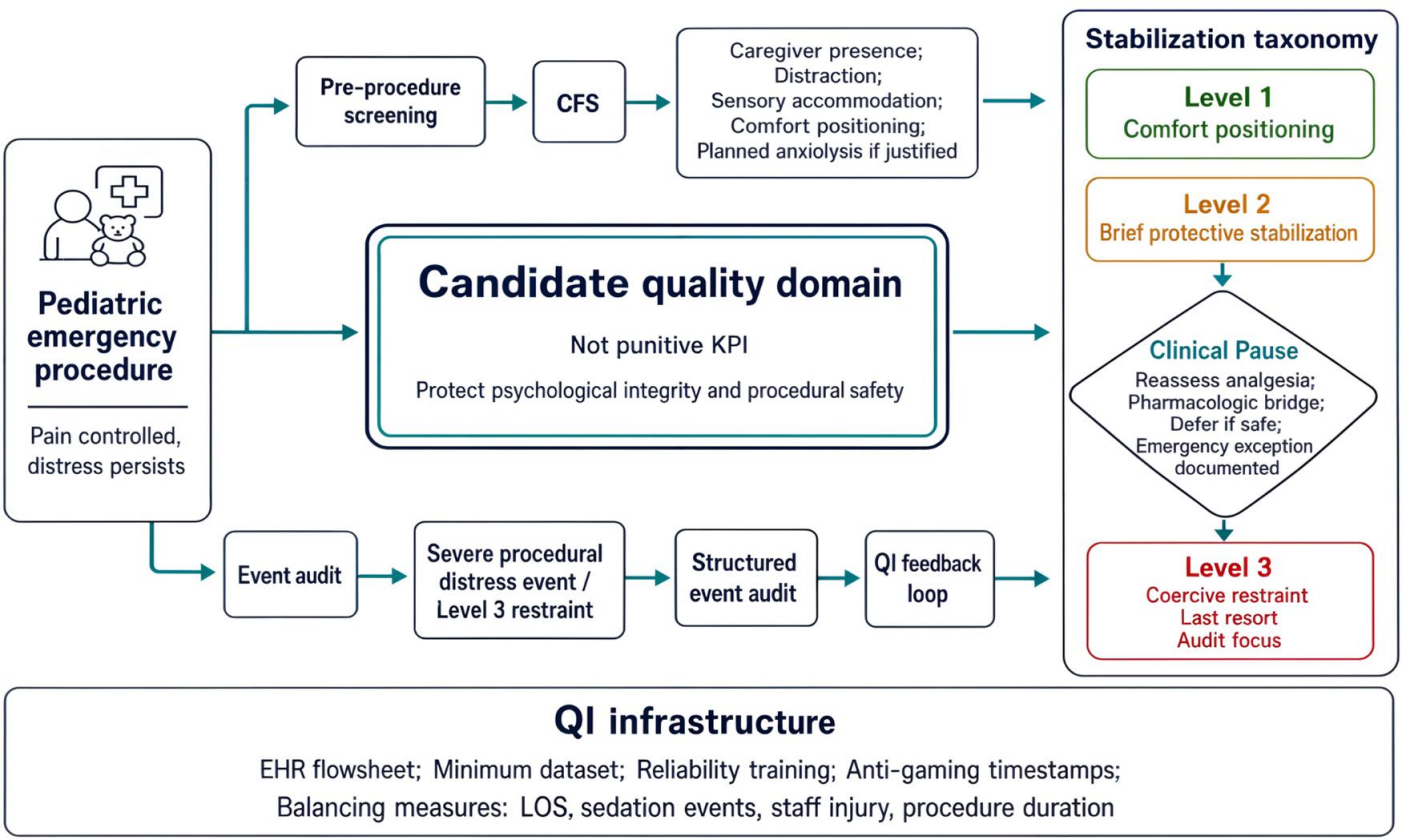
Procedural distress as a candidate quality domain in pediatric emergency medicine.

Although the child perceives no sharp somatic pain, forceful immobilization and loss of autonomy can still create traumatic procedural memories. Adequate local anesthesia does not necessarily prevent fear, loss of control, or traumatic procedural memory. Consequently, evaluating procedural quality solely by the absence of somatic pain is incomplete. Unmanaged procedural distress may contribute to later healthcare avoidance and vaccine hesitancy (24–26). Breaking this cycle requires elevating distress management from an individual clinician's soft skill to a more consistently audited institutional priority. Figure 1 summarizes the proposed two-track framework for operationalizing procedural distress as a candidate quality domain, distinguishing pre-procedure anticipatory fear screening from post-event auditing of severe procedural distress and Level 3 restraint use.

## 2 From subjective observation to operational metric

To systematically address this clinical gap and translate procedural distress from a declarative ethical concept into an auditable quality metric, its semantic and operational boundaries must be rigorously established. In the chaotic ED setting, acute nociception and psychological anxiety are rarely mutually exclusive; rather, they frequently coexist and bidirectionally amplify one another. However, previous conceptualizations of “procedural distress” have often been overly descriptive, relying on subjective clinical terminology such as “severe,” “inconsolable,” or “disproportionate" (4). These subjective descriptors are notoriously difficult to encode consistently, rendering them insufficient for quality improvement (QI) audits, retrospective chart reviews, or inter-institutional benchmarking.

### 2.1 Differentiating the anticipatory state from the adverse event

To bridge this gap, clinicians increasingly utilize anxiety-specific scales like the Children's Fear Scale (CFS). However, utilizing the CFS as a retrospective proxy for a “distress event” represents a methodological vulnerability. The CFS measures an internal state of anticipatory fear, which is critical for prospective risk stratification, but it does not capture the operational severity of a behavioral breakdown. Conflating the screening of an emotional precursor with the occurrence of an adverse event undermines QI accuracy. Therefore, a robust QI framework must utilize a bifurcated approach: (1) Tools like the CFS should be deployed at triage or immediately pre-procedure. Identifying high anticipatory anxiety early allows clinicians to proactively mobilize Child Life specialists, employ targeted distraction, or utilize bridging anxiolytics (5). (2) Institutions must implement an independent, objective logging system—such as a dedicated module in the nursing Electronic Health Record (EHR) flowsheet—specifically designed to capture procedural distress as an operational failure, completely separate from the initial anxiety score.

### 2.2 Establishing actionable thresholds for quality auditing

If procedural distress is to be treated as a candidate quality domain, it requires strict operationalization based on measurable triggers. We propose defining a “severe procedural distress event” not merely as a transient display of pediatric apprehension, but as an acute state of multidimensional behavioral dysregulation that severely impedes the delivery of care. To ensure inter-rater reliability and consistent coding, a reportable Distress Event is objectively triggered when a procedural encounter meets at least one of the following thresholds: (1) Continuous, unmanageable physical resistance necessitating the unplanned interruption, suspension, or complete abandonment of a procedure for greater than a predefined limit (e.g., >3 min) specifically for behavioral de-escalation. (2) The sudden, unplanned requirement of two or more additional personnel (excluding parents/caregivers) specifically mobilized for the purpose of forced physical stabilization or restraint to overcome active resistance. (3) The reactive, unplanned escalation to systemic pharmacological sedation or anxiolysis due to severe behavioral non-compliance, after an initial non-pharmacological or local-only strategy has failed.

Establishing these hard thresholds allows auditors to differentiate true procedural distress from normal emotional expression (e.g., brief, consolable crying) and volitional non-cooperation (developmentally normative opposition devoid of autonomic panic), as the latter rarely cross these resource-intensive boundaries. It also forces the explicit separation of psychological distress from generalized agitation driven by pathological etiologies, such as hypoxia or post-ictal states (6).

For auditing purposes, behavioral dysregulation should not be equated with all crying, protest, or non-cooperation. Brief crying, verbal refusal, withdrawal of a limb, or consolable resistance may be developmentally expected and should not automatically be coded as a severe distress event. A reportable event requires sustained loss of behavioral organization that materially disrupts care, such as prolonged interruption, unplanned mobilization of additional staff for forced stabilization, or reactive escalation to systemic anxiolysis or sedation. The framework also excludes agitation primarily attributable to medical pathology. Hypoxia, hypoglycemia, delirium, intoxication, head injury, and post-ictal states may produce agitation that resembles procedural distress but requires a different clinical response. These encounters should be coded separately or excluded from distress-event denominators unless the procedural component can be clearly distinguished.

## 3 The neurodivergent imperative and the ethics of immobilization

This conceptual reframing must explicitly encompass a rapidly growing and highly vulnerable demographic: neurodivergent pediatric patients. This population includes children with autism spectrum disorder (ASD), attention-deficit/hyperactivity disorder (ADHD), and varied sensory processing differences (7). The standard ED environment is inherently hostile to sensory regulation. Unpredictable acoustic alarms, high-intensity lighting, prolonged waiting times, and unfamiliar tactile inputs frequently precipitate severe sensory overload for these patients, resulting in behavioral meltdowns that are neurologically distinct from standard anticipatory fear (8).

In these populations, the routine deployment of coercive physical restraint poses exceptional psychological and physical risks. When a neurodivergent child experiences a sensory meltdown, physical immobilization compounds the neurological overload, dramatically increasing the risk of mechanical injury, severely eroding trust, and cementing traumatic medical memories (9). Consequently, unmanaged distress often establishes a negative behavioral baseline, dictating the need for deep, high-risk pharmacological sedation for all future, even minor, healthcare encounters (10).

### 3.1 A tiered taxonomy of physical stabilization

A major barrier to auditing restraint as a QI metric is the ambiguity of terminology. Different institutions, and even different practitioners within the same ED, vary wildly in how they classify “parental assistance,” “comfort positioning,” and “papoosing.” Broadly categorizing all holding as “restraint” creates vast data inconsistencies, prevents accurate benchmarking, and risks alienating frontline clinicians who routinely use gentle guidance. The total abolition of physical stabilization is neither clinically feasible nor ethically sound. In time-critical emergencies (e.g., active airway compromise), or during delicate procedures where sudden movement risks catastrophic injury (e.g., facial laceration repair proximal to the globe), brief physical stabilization remains a necessary protective measure.

To achieve standardized auditing, we propose a three-tiered taxonomy aligned with current regulatory and pediatric nursing documentation systems. **The baseline, Level 1 (Comfort Positioning), involves non-coercive,** parent- or staff-assisted, upright, or semi-upright positions (e.g., chest-to-chest sitting, straddling). The child is securely held to provide proprioceptive comfort and prevent sudden jerks, but the positioning does not forcefully override the child's active, continuous physical resistance. This should be documented as standard trauma-informed care, not an adverse event. **If specific risks emerge, clinicians may escalate to Level 2 (Brief Protective Stabilization),** a targeted, time-limited immobilization of a specific body part (e.g., holding a forearm steady during venipuncture) necessary to prevent imminent mechanical injury during an anatomically delicate intervention. The intent is pure risk-minimization, and the duration is explicitly brief (e.g., <3 min). **At the most restrictive end of the spectrum, Level 3 (Coercive Restraint)** Encompasses the forceful, sustained immobilization of a patient—whether supine, lateral, or seated—against their active, violent, and sustained physical resistance. This frequently requires multiple staff members, body weight compression, or mechanical restricting devices (e.g., papoose boards).

Level 2 protective stabilization is narrow, brief, and anatomically targeted. It is used to prevent immediate mechanical injury during a specific procedural step, such as steadying a forearm during venipuncture or limiting movement near the eye during laceration repair. Level 3 coercive restraint is broader and more sustained. It involves forceful immobilization against active, continuing resistance and often requires multiple staff members, body-weight pressure, or a restrictive device. These distinctions should be supported by staff calibration using standardized scenarios, because no taxonomy can be fully reliable without shared training.

In a QI auditing framework, data extraction must clearly delineate its sources(such as structured nursing flowsheets, sedation records, or specific event reports)and should primarily target Level 3 events. The objective is to transition Level 3 Coercive Restraint from a routine convenience to a heavily scrutinized intervention of last resort, utilized only when alternative de-escalation strategies have unequivocally failed.

Cultural and international variability must also be considered. Expectations regarding parental presence, acceptable holding practices, sedation thresholds, documentation of restraint, and legal oversight differ substantially across health systems. A taxonomy developed in one country should therefore not be treated as universally normative without local adaptation. The value of the proposed tiers lies in creating shared language for comparison and training, while allowing institutions to align implementation with local law, culture, staffing, and family-centered care practices.

### 3.2 The “clinical pause” framework to mitigate moral injury

To translate these ethical stances into actionable operational steps, EDs should institutionalize the “Clinical Pause.” Advocating for distress reduction must not serve as a moral indictment of front-line clinicians, who frequently operate under severe systemic pressures. Repeatedly participating in the forceful immobilization of a terrified child contradicts foundational tenets of non-maleficence, contributing to compassion fatigue and professional burnout (11).

The Clinical Pause replaces moral distress with a safe, protocolized decision-making framework. When a procedure escalates toward a Level 3 restraint scenario, the clinical team must be systematically empowered by institutional policy to temporarily halt the intervention. Except in life-saving emergencies, this structured timeout serves as a critical decision node. It prompts the team to evaluate whether the current analgesic strategy has failed, whether targeted pharmacological bridging (e.g., intranasal anxiolytics) is required, or whether the procedure can be safely deferred. By converting an ethical dilemma into an objective clinical algorithm, leadership protects both the patient's autonomy and the clinician's psychological safety.

## 4 The operational efficiency hypothesis

When advocating for the institutionalization of trauma-informed care and distress management, a frequent and formidable barrier cited by hospital leadership is the “time paradox"—the assumption that prioritizing psychological comfort (e.g., waiting for topical anesthetics or deploying Child Life specialists) inherently exacerbates chronic ED overcrowding and prolongs Length of Stay (LOS). In response, proponents often argue that unmanaged distress may actually act as a primary, hidden driver of operational bottlenecks. To maintain scientific rigor and establish distress as a credible quality domain, it is crucial to clearly distinguish robust empirical data from mechanistically plausible inferences.

We propose an “efficiency chain” as a hypothesis-generating model rather than an established causal pathway. Table 1 summarizes the proposed theoretical model and evidence grading for the procedural distress–inefficiency chain. The model describes plausible mechanisms through which severe autonomic arousal and behavioral resistance may disrupt clinical workflows, including vascular access attempts and specimen collection. The strength of evidence varies across links in this chain, and prospective studies are required before these relationships can be treated as causal or generalizable quality indicators.

**Table 1:** Theoretical model and evidence grading for the procedural distress-inefficiency chain.

Clinical linkage	Proposed mechanistic hypothesis	Nature of evidence & future validation needs
Distress & procedure duration	Active behavioral resistance necessitates procedural interruption, prolonged de-escalation efforts, and the gathering of additional staff for physical stabilization.	Strong Observational. Consistent data link high pre-procedural anxiety and non-cooperation to significantly increased total procedure times and higher human resource utilization.
Distress & PIVC first-pass success	Catecholamine surge → sympathetic peripheral vasoconstriction → acute decrease in vein caliber and visibility → higher likelihood of needle misplacement.	Theoretical/Mechanistically Plausible. Supported by clinical correlations between agitation and PIVC failure. *Validation Pathway:* Prospective cohort studies utilizing point-of-care ultrasound to measure dynamic changes in venous cross-sectional area in response to escalating CFS scores, controlling for age, dehydration, and operator experience.
Thrashing & specimen hemolysis	Mechanical shear forces from thrashing, combined with excess vacuum pressure applied to constricted veins, physically traumatize erythrocytes during collection.	Moderate *in vitro*/Extrapolated. Strong *in vitro* evidence confirms mechanical shear causes hemolysis. *Validation Pathway:* Large-scale clinical studies are needed to isolate the specific attributable fraction of behavioral thrashing to systemic ED hemolysis rates, rigorously controlling for sampling technique and gauge size.

## 5 A quality improvement framework for procedural distress

Translating procedural distress from an abstract ethical concern into an actionable, measurable entity requires its formal integration into the emergency department's core quality improvement (QI) infrastructure. However, asserting that distress reduction or the minimization of coercive restraint should instantly become a mandatory “Key Performance Indicator (KPI)” remains an empty, and potentially dangerous, proposition without rigorous operational definitions. In the high-stakes, resource-constrained environment of acute care, poorly operationalized KPIs frequently fall victim to Goodhart's Law: when a measure becomes a target, it ceases to be a good measure (12). Rigid KPIs tied to departmental funding or individual performance evaluations risk unfair auditing, statistical incomparability across providers, staff resentment, and ultimately, the clinical gaming of data (13).

Instead of punitive KPIs, we propose framing these metrics as Candidate Quality Domains. A robust QI initiative must establish clear denominators, specify uniform data sources, and, crucially, monitor balancing measures. Balancing measures ensure that systemic efforts to reduce physical restraint do not inadvertently cause parallel harm, such as unacceptable delays in time-sensitive therapies or inappropriate escalations in deep procedural sedation. To serve as a blueprint for institutional implementation, we propose a minimum viable dataset for auditing procedural distress (Table 2).

**Table 2:** Proposed minimum dataset for quality auditing of procedural distress.

Metric category	Operational definition (numerator/denominator)	Safety exclusions & anti-gaming rules	Balancing measures (to monitor unintended consequences)
Process measure: preprocedural screening rate	Numerator: Eligible procedures with a documented, validated anxiety score (e.g., CFS) prior to the first attempt. Denominator: Total eligible non-emergent invasive procedures.	Strict Exclusions: Triage Level 1 (active resuscitation), altered mental status, or immediate life-saving interventions. Rule: Assessment must occur prior to patient positioning.	Time-to-Provider Evaluation: Monitor to ensure screening protocols do not create systemic triage or operational bottlenecks.
Outcome measure: level 3 coercive restraint rate	Numerator: Procedures utilizing Level 3 Coercive Restraint (forceful, sustained immobilization against active resistance). Denominator: Total non-emergent procedures performed.	Strict Exclusions: Airway management, massive hemorrhage control, cervical spine clearance, or anatomically delicate interventions proximal to the globe.	Procedure Duration & Staff Injury Rates: Monitor if avoiding restraint unsafely prolongs care or compromises provider safety due to sudden movement.
Outcome measure: reactive rescue sedation rate	Numerator: Procedures requiring unplanned escalation to systemic sedation/anxiolysis due to severe behavioral non-compliance. Denominator: Total procedures initiated with only local/topical anesthesia or non-pharmacological support.	Anti-Gaming Rule: Proactive sedation plans *must be documented and clinically justified prior to the first procedural attempt*. Retrospective reclassification is audited as a failure.	Adverse Sedation Events & LOS: Monitor for the overutilization of pharmacological agents, respiratory depression, and prolonged ED Length of Stay (LOS).

CFS, Children's Fear Scale; LOS, length of stay. This framework aligns with standard QI methodologies to ensure safe, measurable, and reliable implementation.

Implementation should be staged according to institutional resources. A tertiary pediatric ED may be able to integrate a dedicated Procedural Experience Flowsheet, routine inter-rater reliability training, and formal Child Life escalation pathways. In contrast, a community hospital or lower-resource ED may begin with a smaller bundle: documenting whether a procedure required unplanned additional staff, whether sustained coercive restraint occurred, and whether anxiolysis or sedation was escalated after a failed initial attempt. These data elements can be embedded into existing nursing procedure notes rather than introduced as a separate documentation system. Training can likewise begin with brief case-based calibration during existing staff meetings or simulation sessions, rather than a new stand-alone curriculum.

### 5.1 Data sources and consistency training

For metrics to be reliable, they cannot depend on retrospective, free-text narrative notes, which are notoriously prone to recall bias and subjective sanitization (e.g., documenting “patient was uncooperative” instead of “required four staff members for forced immobilization”). Data must be extracted from structured, mandatory fields within the Electronic Health Record (EHR) (14). We recommend integrating a “Procedural Experience Flowsheet” into the standard pediatric nursing documentation. This flowsheet should utilize a drop-down taxonomy aligned with the stabilization tiers defined previously. To ensure data integrity, institutions must mandate brief, routine inter-rater reliability training for pediatric nursing staff, utilizing standardized simulation scenarios to calibrate the coding of distress events and restraint levels (15).

For bedside use, these definitions should be translated into short structured fields rather than reproduced as lengthy narrative criteria. A practical flowsheet might ask whether sustained coercive restraint occurred, whether additional staff were unexpectedly required for stabilization, whether the procedure was paused for behavioral de-escalation, and whether systemic anxiolysis or sedation was escalated after an initial failed attempt. The goal is not to impose a uniform tertiary-center model across all emergency settings. A pragmatic implementation pathway should preserve the core definitions while allowing local variation in staffing, EHR capacity, sedation resources, and access to Child Life specialists. Community hospitals may focus initially on identifying high-risk encounters and reducing sustained coercive restraint, whereas tertiary pediatric centers may be positioned to evaluate broader process and outcome measures.

### 5.2 Establishing “anti-gaming” data rules

A recognized risk in establishing performance metrics is the phenomenon of “metric gaming"—where clinical documentation is retroactively altered or loosely interpreted to artificially inflate compliance (16). In the context of procedural distress, the boundary between “planned, proactive sedation” and “reactive rescue sedation” is highly vulnerable to manipulation. If an institution tracks “reactive rescue sedation” as a failure of distress management, a clinician might be incentivized to classify a sedation event that occurred after an initial failed, traumatic restraint attempt as a “planned” intervention in the patient's chart (17).

A distress quality domain must also be protected from unintended harm. If implemented poorly, it could increase documentation fatigue, create medicolegal anxiety, or make clinicians hesitate before performing necessary urgent interventions. For this reason, the framework should be explicitly non-punitive and system-facing. Documentation should rely on a small number of structured fields rather than long free-text narratives, and audit results should be used to identify workflow gaps, training needs, and resource constraints rather than to assign individual blame. Psychological comfort must be balanced with procedural urgency. The Clinical Pause is not intended to apply during resuscitation, airway compromise, uncontrolled bleeding, or other time-critical scenarios in which delay would create greater harm. In non-emergent procedures, however, a brief pause may prevent escalation to sustained coercive restraint, repeated failed attempts, or reactive sedation. Balancing measures such as time to critical intervention, procedure completion rate, staff injury, adverse sedation events, and ED length of stay should be monitored alongside restraint and distress metrics.

To prevent this, institutions must institute strict data-governance rules hardwired into the EHR metadata. First, under the Timestamp Criterion,to be classified and excluded as “Planned Sedation,” the sedation order, the specific pharmacological agent, and the clinical rationale (e.g., “documented history of severe procedural trauma,” “Level 3 ASD with known tactile defensiveness”) must be formally timestamped in the EHR prior to the initiation of any physical procedural preparation; **Second, according to the Attempt Criterion,** any systemic anxiolytic or sedative ordered after a documented first procedural attempt has failed, or after the patient has reached a state of autonomic hyperarousal, is automatically coded as a “Reactive Rescue Event,” regardless of the subsequent narrative documentation.

## 6 Bridging the gap via safe pharmacological anxiolysis rather than chemical restraint

While non-pharmacological interventions—orchestrated by Child Life specialists, incorporating age-appropriate distraction, and utilizing comfort positioning—remain the absolute foundation of trauma-informed care, they possess an inherent efficacy ceiling. For highly anxious phenotypes, severely neurodivergent populations, or previously traumatized children, behavioral de-escalation alone is frequently insufficient to overcome the acute sympathetic surge associated with an invasive procedure. In these scenarios, modern EDs must possess an armamentarium of robust pharmacological bridging therapies (10).

Pharmacological anxiolysis should be treated as a bridge, not as a substitute for trauma-informed care. Environmental modification, sensory accommodation, caregiver presence, developmentally appropriate preparation, and comfort positioning remain the first-line infrastructure for reducing distress. Medication becomes appropriate when these measures are insufficient for a specific child or procedure, or when prior history indicates a high risk of severe dysregulation despite optimized non-pharmacological support.

Advocating for the increased use of pharmacological agents to reduce physical restraint carries an inherent ethical risk: the inadvertent promotion of “chemical restraint.” Administering deep sedatives merely as a substitute for systemic environmental accommodations, or to compensate for a lack of child life resources, violates the principle of minimal effective intervention (9). Replacing physical coercion with unmonitored pharmacological over-sedation simply trades mechanical risk for airway risk (18). Therefore, the utilization of bridging agents must strictly adhere to authoritative institutional safety boundaries.

Institutional governance is essential. Use of intranasal dexmedetomidine, nitrous oxide, or other anxiolytic strategies should occur under locally approved protocols that define eligibility, contraindications, monitoring requirements, staff credentialing, rescue capacity, discharge criteria, and documentation standards. Without these safeguards, pharmacological anxiolysis risks drifting toward chemical restraint rather than functioning as part of a proportionate, patient-centered procedural plan.

### 6.1 Intranasal dexmedetomidine (iN Dex)

Dexmedetomidine, a highly selective $\alpha_{2}$ -adrenergic agonist, has revolutionized pediatric procedural anxiolysis. Administered via the mucosal route (typically at doses of 2 to 3 mcg/kg), it produces a state of cooperative sedation and anxiolysis that closely mimics natural non-REM sleep, crucially without the significant respiratory depression characteristic of opioids or benzodiazepines (19).

Regarding operational caveats, it is important to recognize that while its safety profile is excellent, IN Dex is not a panacea. Its primary operational limitation is its pharmacokinetic onset time, typically requiring 25 to 45 min to achieve peak clinical effect (20). In a crowded ED, this delay is frequently cited as a barrier; however, clinicians must be institutionally supported to allow this medication the necessary time to work. Rushing the procedure at minute 15 almost guarantees failure, triggering the cascade back to physical restraint.

From a safety boundaries perspective, while respiratory drive is maintained, dexmedetomidine can induce transient bradycardia and hypotension. Consequently, institutional guidelines must mandate its use only in environments equipped with continuous cardiopulmonary monitoring (ECG and pulse oximetry) and staffed by clinicians credentialed in pediatric advanced life support and airway rescue (18).

### 6.2 Inhaled nitrous oxide

Nitrous oxide (typically delivered at a 50% to 70% concentration blended with oxygen) offers potent (21), rapid-onset anxiolysis and mild analgesia, with the distinct advantage of an equally rapid offset once the gas is discontinued. This pharmacokinetic profile aligns perfectly with the rapid throughput demands of the pediatric ED (22).

There are several operational caveats to its use, most notably that successful administration requires a baseline level of patient cooperation to maintain a seal on the delivery mask or mouthpiece. Therefore, it is frequently ineffective in deeply dysregulated toddlers or children experiencing severe sensory meltdowns who actively and forcefully reject the mask (23).

In terms of safety boundaries, administration requires specialized scavenging systems to protect occupational health. Furthermore, prolonged administration or combining with other systemic sedatives exponentially increases the risk of transitioning the patient from minimal anxiolysis into deep sedation or general anesthesia. This necessitates strict adherence to fasting guidelines and continuous clinical vigilance by the attending team (18).

## 7 Multicenter validation and implementation

While the ethical imperative to manage procedural distress is undeniable, the assertion that formalizing it as an audited quality domain will uniformly improve operational efficiency requires rigorous empirical validation. Currently, the evidence base leans heavily on mechanistic hypotheses and single-center QI initiatives.

To bridge this gap, the pediatric emergency medicine community must prioritize large-scale, multicenter trials (e.g., stepped-wedge cluster randomized designs). These future models must explicitly investigate whether systematic auditing and structured “Clinical Pause” frameworks reliably reduce coercive restraint and improve operational workflows (e.g., increased first-pass success, decreased hemolysis) without inadvertently worsening balancing measures like overall ED length of stay or adverse sedation events. By rigorously controlling for clinical confounders—such as patient age, baseline dehydration, and operator experience—researchers can definitively validate these hypotheses, providing administrators with the evidence-based mandate required to fund comprehensive trauma-informed care programs.

The associations proposed in this Perspective should therefore be interpreted as testable hypotheses. Future studies should examine whether distress auditing and structured clinical pause protocols reduce coercive restraint or improve procedural workflow after adjustment for patient age, dehydration, baseline neurodevelopmental status, procedure type, operator experience, and ED crowding.

## 8 Limitations

This Perspective has several important limitations. First, it does not present original empirical data. The proposed quality framework is therefore conceptual and should be regarded as a basis for future measurement development and implementation research rather than as a validated standard of care. Second, several proposed associations, particularly those linking procedural distress to vascular access failure, hemolysis, and ED throughput, remain mechanistically plausible but incompletely tested in clinical settings. Third, the proposed definitions and thresholds require multicenter validation before they can support benchmarking across institutions. Fourth, emergency departments vary widely in staffing, EHR infrastructure, sedation capacity, Child Life availability, legal requirements, and cultural norms surrounding restraint and family participation. A framework that is feasible in a tertiary pediatric center may require substantial adaptation in a community hospital or lower-resource setting. Finally, standardizing distress measurement is intrinsically difficult because procedural behavior reflects age, developmental stage, neurodivergence, prior medical trauma, caregiver presence, medical acuity, and procedural urgency. The thresholds proposed here are intended to improve reproducibility, but they will require training, reliability testing, and iterative refinement.

## 9 Conclusion

The true measure of quality in pediatric emergency care extends beyond the biological resolution of acute pathology; it must holistically encompass the preservation of a child's psychological integrity. Having successfully established somatic pain management as a fundamental standard of care, the medical community must now apply that same systemic rigor to mitigating procedural distress. By reframing procedural distress from an accepted operational byproduct to a measurable, preventable adverse event, healthcare systems can transition away from the routine use of coercive physical restraint. Ultimately, treating procedural distress as a core quality domain ensures that the life-saving physical interventions provided today do not inflict the psychological trauma our vulnerable patients are forced to carry into tomorrow.

## Data Availability

The original contributions presented in the study are included in the article/Supplementary Material, further inquiries can be directed to the corresponding author.
